# Self-reported sexual health: Breast cancer survivors compared to women from the general population – an observational study

**DOI:** 10.1186/s12885-017-3580-2

**Published:** 2017-08-30

**Authors:** Anne Oberguggenberger, Caroline Martini, Nathalie Huber, Lesley Fallowfield, Michael Hubalek, Martin Daniaux, Barbara Sperner-Unterweger, Bernhard Holzner, Monika Sztankay, Eva Gamper, Verena Meraner

**Affiliations:** 1 Department of Psychiatry, Psychotherapy and Psychosomatics, Medical University of Innsbruck, Christoph-Probst-Platz 1, Innrain 52, 6020 Innsbruck, Austria; 2 Sussex Health Outcomes, Research & Education in Cancer (SHORE-C), University of Sussex, Brighton, BN1 9PX UK; 3 Breast Center, County Hospital Schwaz, Swarovskistraße 1-3, 6130 Schwaz, Austria; 4 Department of Radiology, Innsbruck Medical University, Anichstraße 35, 6020 Innsbruck, Austria

**Keywords:** Breast neoplasms, Sexuality, Survivorship, Self report

## Abstract

**Background:**

Cancer survivorship is of increasing importance in post-treatment care. Sexual health (SH) and femininity can be crucial issues for women surviving cancer. We aimed to determine a more complete understanding of the contribution that a breast cancer (BC) diagnosis and its treatment exert on patients’ follow-up SH. For this purpose, self-reported levels and predictors of SH in breast cancer survivors (BCS) were compared with those of women with no previous or current BC (WNBC).

**Methods:**

BCS and WNBC underwent a comprehensive, cross-sectional patient-reported outcome (PRO) assessment. Validated PRO instruments were used to measure SH, body image, anxiety and depression and menopausal symptoms. Assessments were performed within the routine clinical setting.

Instruments used were the Sexual Interest and Desire Inventory - Female, Sexual Activity Questionnaire, Body Image Scale, Hospital Anxiety and Depression Scale and the Menopause-Specific Quality of Life Questionnaire.

**Results:**

One hundred five BCS (average time since diagnosis of 3 years) and 97 WNBC with a mean age of 49 years completed the assessment. SH was significantly worse in BCS compared to WNBC (*p* = 0.005; BCS SIDI-F mean = 24.9 vs. WNBC mean = 29.8). 68.8% of BCS and 58.8% of WNBC met criteria of a hypo-active sexual desire disorder. Higher depressive symptoms, higher age and lower partnership satisfaction were predictive for poorer SH in BCS.

**Conclusion:**

SH problems are apparent in BCS and differ significantly from those seen in the general population. Consequently, BC survivorship care should include interventions to ameliorate sexual dysfunction and provide help with depressive symptoms and partnership problems, which are associated with poor BCS SH.

## Background

A decrease in breast cancer (BC) mortality and improved screening and treatment options has lead to a steadily increasing group of breast cancer survivors (BCS), which in turn create new demands in survivorship health care [1–5]. Quality of life (QoL) issues are of high relevance in the after-care of BC patients [6, 7]. Breast cancer and its associated treatments are often linked to a number of physical and psychosocial changes and uncertainties that may have a deleterious impact on partnership and sexuality. Though several studies indicate that a majority of BCS show overall QoL scores comparable to those of the general population [8], adverse effects from cancer treatment can continue to impact upon sexual health (SH) for years [9–11]. With a prevalence of 23–85%, sexual morbidity is amongst the most frequent side effects and consequences of a BC diagnosis and associated treatments [12]. Sexual morbidity encompasses a wide range of problems and symptoms including lack of sexual desire and interest, body satisfaction, frequency of intercourse, sexual satisfaction, arousal, orgasm, and pain associated with intercourse [13]. Levels of these sexual problems seem to exceed those of women with no previous or current BC (WNBC) in the same age range [6, 8]. Moreover, adverse sexual effects have been illustrated to be associated with worse cancer-related distress, depression, symptom severity and overall QoL [14–16].

Despite an increasing research interest in the relative contribution of a BC diagnosis to sexual problems in the long-term, research on the persistence of the well known disease- and treatment-related sexual adverse effects into survivorship has received relatively little attention. We currently lack data on this subject systematically assessed in a routine clinical setting as data derived from clinical trials does not usually include this topic. Patients’ subjective perspectives, assessed routinely provide complementary information contributing to a better understanding of disease- and treatment-related dysfunction particularly within sensitive domains such as sexuality. Validated patient-reported outcome (PRO) measures provide an efficient option for a more comprehensive assessment of SH impairments. Moreover, gathering patient reported information on sexual problems in BCS can help to improve the detection rates of sexual adverse effects and therefore make them amenable to individualized clinical care efforts in daily clinical practice [17]. This might result in a reduction of sexual problems and subsequently an improvement of overall QoL in BCS.

## Aims

We investigated the self-reported SH outcome of BCS in routine after-care in comparison to WNBC. Additionally, predictors of SH were investigated. In detail, we addressed the following research questions: (1) Does self-reported SH of BCS differ from those of WNBC? (2) Is the SH of BCS predicted by treatment-related and clinical variables? (3) Which self-reported femininity issues, psychosocial issues and sociodemographic variables predict follow-up SH?

## Methods

### Sample

#### Breast cancer survivors

Inclusion criteria for women in the BCS group were the following: Breast cancer patients who (1) had a confirmed diagnosis of BC, (2) were off primary treatment, (3) had no disease recurrence, (4) were aged 18 years or older, (5) were fluent in German, and (6) had no overt cognitive impairment. Clinical data of the BCS group are presented in Table 1.

**Table 1:** BCS’ clinical characteristics

		Breast cancer patients *N* = 105%
Time since diagnosis	Mean (SD)	3.2 (2.2) years
	Range	0.3-17 years
Grading (TNM)	Grade I	16.3%
	Grade II	60%
	Grade III	23.8%
Primary surgical treatment	Breast conserving surgery	66.7%
	Mastectomy	33.3%
Endocrine treatment^a^		64.6%
Current endocrine treatment^b^		53.6%
Radiotherapy		73.5%
Chemotherapy		49.5%
Menopausal state	Premenopausal	57.7%

^a^endocrine treatment received

^b^Ongoing endocrine treatment at assessment time point

Reference sample of women with no previous or current breast cancer.

For the purpose of comparison, a sample of WNBC without a history of cancer, who were comparable to the BCS sample regarding age and education were included in the study. This reference sample was approached at the Department of Radiology, Medical University of Innsbruck. It comprised women attending the routine screening or any other mammography. Exclusion criteria were, beside a previous cancer disease, no suspection of BC as well as the participation at a high risk screening due to a highly positive family history for BC or a confirmed BRCA1 or BRCA 2 mutation.

#### Procedure

The study was designed as a cross-sectional PRO survey implemented in routine clinical after-care at the Department of Gynecology and Obstetrics, Medical University of Innsbruck. This includes a consecutive approach of eligible patients presenting at the Department’s outpatient clinic for one of their routine after-care check-up. Searching the clinic’s medical records up-front identified eligible patients. Patients were approached at their routine after-care check-up by their treating physician and invited to participate in the study. The invitation included a short explanation of the study up-front. If patients were interested, full study informed consent was gathered by the treating physician. Following written informed consent, patients completed a comprehensive PRO assessment focussed upon SH, body image, menopausal symptoms and psychological distress. Menopausal state was assessed dichotomously (pre- vs. postmenopausal) as reported in the patient’s medical history. Details on the PRO questionnaires are given below. Patients were given the opportunity to complete the assessment semi-anonymously (including only clinical data, no name).

WNBC presenting at Department of Radiology, Medical University of Innsbruck for their routine or any other mammography screening for BC were randomly and consecutively approached in accordance with the matching criteria to the BCS sample (age and education). After the mammography screening confirming the absence of a BC diagnosis women were approached and invited to join the study. Consenting participants provided written informed consent. WNBC completed the same PRO survey as the BCS sample with some disease-related questions being adapted. The survey also included sociodemographic information and information on womens’ medical history (other chronic disease and current medication intake).

#### Main outcome measures

Sexual Activity Questionnaire (SAQ).

The SAQ is a reliable and validated short self-report measure for the assessment of female sexuality in BC patients [18]. It is composed of 3 sections: items of section 1 contribute to the differentiation of sexual active and inactive women. In section 2 reasons for sexual inactivity are assessed. The third section targets on SH only in sexually active women. Ten items assess pleasure, discomfort with intercourse and habit. The response format is a 4-point Likert scale with high values indicating high sexual function.

#### Sexual interest and desire inventory–female (SIDI-F)

The SIDI-F is a well-validated diagnostic instrument for the determination of women’s hypo-sexual desire disorder (HSDD) [19]. It is composed of 13 items targeting on the following issues: sexual desire, sexual dysfunction, sexual behaviour, and sexual relationship. Additional 4 items evaluate some background information on psychological and physical health issues (partnership satisfaction, negative thoughts, pain, and mood) in order to better understand the patient’s overall sexual functioning but do not contribute to the total SIDI-F scoring. Low scores indicate low sexual functioning. A cut-off of 33 or lower indicates HSDD. For the purpose of this study, a self-report version was developed to provide anonymity particularly for WNBC as well as due to logistic reasons for the data assessment. Response format and scoring system corresponded to the original proxy-rating version (cut-off of ≤33 for HSDD). Psychometric properties of the self-report version were very satisfactory showing a high correlation with the SAQ (*r* = .85, *p* < .001) and very good internal consistency (alpha = .925).

#### Menopause-specific quality of life questionnaire (MENQOL)

The MENQOL was developed as a self-report instrument in order to assess QoL for the menopause [20, 21]. It consists of 29 items composing the subscales vasomotor, psychosocial, and sexual symptoms. No overall sum score is obtained from the questionnaire. Patients are first asked to indicate the presence of a symptom and – if present – its severity on a 7-point Likert Scale. High values indicate high symptoms. The instrument shows good psychometric properties. The MENQOL has also been validated for use in breast cancer survivors (potentially) experiencing menopausal symptoms due to cancer treatment (endocrine treatment, chemotherapy, etc.) ^22^. Menopausal state was recorded in addition.

#### Body image scale (BIS)

Hopwood and colleagues [22] developed the BIS as a PRO measure in collaboration with the European Organisation of Research and Treatment of Cancer Quality of Life study group for the purpose of assessing body image in cancer patients. It is a well-validated, 10 items short instrument suitable for use in clinical trials. The BIS has a single sumscore; the response format is a 4-point Likert scale with high values indicating good body image.

#### Hospital anxiety and depression scale (HADS)

The HADS has been developed as a screening instrument for anxiety and depression in somatically ill patients [23]. It is a 14 items short, self-assessment scale, with 7 items addressing anxiety and depression each. Scores of 8 to 10 indicate moderate levels of anxiety or depression, scores of ≥11 show an anxiety or depressive disorder. Patients rate their symptom severity on a 4-point Likert scale. The instrument shows excellent psychometric properties and is widely used in clinical trials as well as for the purpose of routine screening.

### Statistical analysis

Sample characteristics are presented descriptively using percentages, means, standard deviations, and ranges. Sociodemographic group comparability of BCS and WNBC was verified by use of Chi-square test or independent t-test (two-sided). Group differences between BCS and WNBC with regard to the SH status were analyzed by means of independent t-tests (two-sided). Effect sizes are indicated by Cohen’s d [24]. We performed a linear regression analysis for the investigation of predictors of follow-up SH considering menopausal symptoms, body image, psychological distress, and disease- and treatment-related variables (backward elimination procedure). R^2^ was reported as measure of model determination; b was employed as a measure of effect size in the regression analyses, i.e. beta indicates how many units SH changes per unit increase of the predictor variable.

All analyses were conducted using SPSS 22.

The Ethics Committee of the Medical University of Innsbruck approved the study (study number UN 5240, meeting number 329/4.21).

## Results

### Patient characteristics

**Fig. 1:**
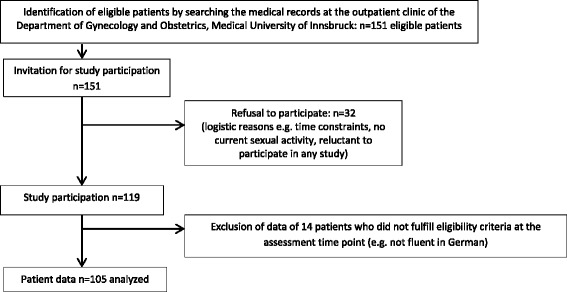
BCS selection and inclusion procedure

**Fig. 2:**
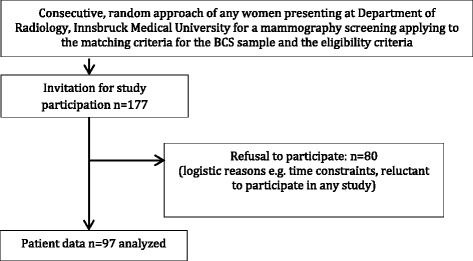
WNBC selection and inclusion procedure

From January to December 2014 patients and WNBC were included in the study. The final BCS group comprised 105 women who were on average 3 years post-diagnosis (0.3 years to 17 years). A reference sample of 97 WNBC was available for the purpose of comparison. Please find details for the selection of participants and inclusion procedure in Fig. 1 and Fig. 2.

In both groups mean age was 49 years (*SD* = 8.7) and 77% had less or equal to 12 years of education, respectively (matching criteria). No group differences were found with regard to all sociodemographic characteristics. Please find further details on the sociodemographic information in Table 2.

**Table 2:** Sociodemographic characteristics of BCS and WNBC

		Breast cancer patients *N* = 105%	WNBC *N* = 97%	Group difference
Age	Mean (SD)	49 years (8.7 years)	49 years (8 years)	matched
	Range	29-70 years	28-70 years	
Age groups	<30	2	2	
	30-40	8.6	8.2	
	41-50	37	39	
	51-60	41	42	
	>60	11.4	8.2	
Marital status	Single	17.3%	7.2%	*p* = 0.057
	Partnership, marriage	76%	88.7%	
	Divorced, separated	6.7%	4.1%	
Education	Compulsory school or less	9.6%	7.2%	matched
	Apprenticeship/ professional school	49%	48.5%	
	A-level	19.2%	20.6%	
	University degree	21.2%	22.7%	
	Other	1%	1%	
Employment	Full time	24.3%	34%	*p* = 0.097
	Part time	35.9%	43.3%	
	Unemployed	3.9%	1%	
	Homemaker	7.8%	8.2%	
	Retired	21.4%	9.1%	
	Other	6.8%	4.1%	

### SH outcome in BCS compared to WNBC

Twenty-eight percent of BCS and 20% of WNBC (n.s.) indicated that they were currently sexually inactive (assessed by means of the SAQ). The primary reason for sexual inactivity was not having a partner followed by lack of interest in sex in both groups. 68.8% of BCS and 58.8% of WNBC scored below the SIDI cut-off of 33 indicating a HSDD.

BCS had significantly more SH impairments than WNBC according to the SIDI-F (*p* = 0.005, *t* = −2.85; BCS mean = 24.9 vs. WNBC mean = 29.8). In addition, BCS reported significantly more discomfort with intercourse (SAQ subscale discomfort) than WNBC (*p* = 0.032, *t* = −2.2; BCS mean = 4.4 vs. WNBC mean = 5). For both scales, we found moderate effect sizes of 0.4 (according to Cohen), respectively. No differences were found for the SAQ subscales pleasure (*p* = 0.21, *t* = −1.2) and habit (*p* = 0.91, *t* = −0.11). Please find details in Table 3.

**Table 3:** Differences between BCS and WNBC regarding their SH outcome, presentation of (sub)scale results

	BCS Mean (SD^e^)	WNBC Mean (SD^e^)	T-Test	Difference	ES^c^, p^d^
SIDI-F^a^	24.9 (13)	29.8 (10.74)	−2.85	4.9	0.41, 0.005
SAQ^b –^ discomfort with intercourse	4.4 (1.9)	5 (1.4)	−2.2	0.6	0.36, 0.032
SAQ^b -^ habit	1.8 (0.9)	1.8 (0.9)	−0.11	0	0, 0.9
SAQ^b -^ pleasure	9.5 (4.8)	10.4 (4.6)	−1.26	0.9	0.19, 0.2

^a^SIDI-F

^b^Sexual Activity Questionnaire

^c^Cohen’s effect size

^d^a *p* value below 0.05 was considered significant

^e^Standard deviation

### Impact of treatment-related and clinical variables on SH in BCS

For the purpose of better understanding the follow-up impact of BC disease and treatment on SH outcome, we considered the following disease- and treatment-related variables for the correlation and regression analysis: grading, type of surgical treatment, chemotherapy, radiation, endocrine treatment and time since diagnosis.

We found no association of these variables with the SIDI-F sumscore. Grading was the only variable significantly associated with SAQ-pleasure (*r* = −.351, *p* = .008) and SAQ-habit (*r* = −.358, *p* = .007). In the linear regression analysis, the predictive value of lower grading for pleasure and habit was confirmed explaining 12.3% (pleasure) and 12.8% (habit) of the variance (Table 4).

**Table 4:** Linear regression model on the association of treatment-related and clinical variables on SH in BCS (*n* = 105)

Predictors	SAQ- pleasure R^2^ ^c^ = 12.3%				SAQ- habit R^2^ = 12.8%			
	β^a^	t	p^b^	95% CL Lower-upper bound	β	t	p^b^	95% CL Lower-upper bound
Grading^d^	−2.65	−2.756	*0.008*	−4.6–0.7	−.53	−2.818	*0.007*	−0.9-0.15
Type of surgical treatment	0.130	1.009	.318		.012	.090	.929	
Chemotherapy	−.006	−.041	.967		−.026	−.192	.849	
Radiation	−.039	−.301	.765		−.112	−.865	.391	
Endocrine treatment	−.102	−.744	.460		.135	.992	.326	

^a^beta coefficient

^b^a *p* value below 0.05 was considered significant

^c^R^2:^ explained variance by the model

^d^dichotomized variable (grade 1 vs. higher grades)

italic print indicates significance

CL: 95% confidence interval

### The predictive value of self-reported femininity issues, psychosocial issues and sociodemographic variables on SH in BCS

We investigated the impact of body image, menopausal symptoms, anxiety and depression, satisfaction with partnership, menopausal state, age, marital state, and education as well as time since diagnosis on SH. The following variables were significantly associated with follow-up SH outcome (SIDI-F) in an up-front univariate analysis: depression (HADS *p* < .001, *r* = −.365), menopausal symptoms (MENQOL-psychosocial *p* < .001, *r* = −.380; MENQOL-vasomotor *p* = .028, *r* = −.228; MENQOL-physical *p* = .001, *r* = −.331; MENQOL-sexual *p* < .001, *r* = −.568), age (*p* = .001, *r* = −.335), satisfaction with partnership (SIDI-diagnostic question *p* < .001, *r* = .542).

In the multivariate analysis, the predictive value of higher partnership satisfaction, lower depression and lower age on follow-up SH outcome (SIDI-F) was demonstrated explaining 38.4% of the variance. Please find details for the respective analysis in Table 5. Menopausal symptoms had – though significantly correlated – no predictive value according to this model (the MENQOL sexual domain was a priori not included in the model since it is supposed to assess the similar construct as the dependent variable).

**Table 5:** Linear regression model on the association of self-reported femininity issues, psychosocial issues and sociodemographic variables on SH in BCS (*n* = 105)

Predictors	SIDI-F R^2^ ^e^ = 38.4%			
	β	t	P^f^	95% CL
BIS^a^	−0.064	−0.67	0.505	
MENQOL^b^				
Vasomotor	−004	−0.431	0.668	
Psychosocial	−0.13	−1.028	0.307	
Physical	−0.144	−1.378	0.652	
HADS-depression^c^	−.94	2.7	0.008	−1.646-0.238
HADS-anxiety^c^	0.108	0.948	0.346	
Satisfaction with partnership^d^	3.664	5.2	<0.001	2.27-5.05
Menopausal state (pre- vs. post menpausal)^g^	−0.069	−0.496	0.621	
Age^g^	−.307	−2.1	0.039	−0.585-0.028
Marital state (with vs. without partnership)^g^	−0.17	−1.154	0.124	
Education (less vs. more than compulsory school)^g^	0.072	0.799	0.427	
Time since diagnosis	0.012	0.137	0.892	

^a^Body image scale

^b^Menopause-Specific Quality of Life Questionnaire

^c^Hospital anxiety and Depression Scale

^d^derived from the SIDI-diagnostic question on partnership satisfaction

^e^R^2^: explained variance by the model

^f^ *p* value below 0.05 was considered significant

^g^dichotomous variables

CL: 95% confidence interval

## Discussion

Cancer survivorship issues have become increasingly important in post-treatment care during the past decade. SH and femininity have been identified among the most crucial subjects for women surviving cancer. In this study, we aimed to elaborate and understand more of the relative contribution of a BC diagnosis and its treatment to female SH over time. For this purpose, the consideration of subjective patient data is inevitable. We investigated self-reported levels and predictors of SH outcome in BCS in the clinical routine in comparison to WNBC.

Even years after treatment, BC patients still reported distinct levels of sexual health impairments that differed significantly from that of women without a history of BC. BCS were not only more frequently sexually inactive, but also met the criteria for a HSDD more often. Almost 70% of BCS qualified for a HSDD based on self-reports which is comparable to results from a recent study from Raggio and colleagues [25] who found rates of even 77% in BCS using the FSFI as outcome measure. Similar findings were reported by Panjari and colleagues [10] who observed sexual functioning problems in 70% of BC patients up to 12 months post-diagnosis.

Overall, BCS reported worse SH than WNBC (according to the SIDI-F). This result complements previous findings of higher sexual dysfunction observed in BC patients short after treatment [17, 26–28]. Our results confirm some previous evidence. Already15 years ago, Dorval and colleagues [29] reported that BCS did not differ from population controls in all QoL domains except sexuality, which was worse in BC patients. However, despite the invention of new treatment regimes and treatment efforts SH impairments still seem to be a major problem related to BC. Only recently, Boquiren and colleagues [30] illustrated that BCS experienced poorer sexual functioning than the female general population. Corresponding results have been obtained by Bredart and colleagues [31]. Particularly, the issue of discomfort with intercourse seems to be a major factor contributing to this difference. Quite surprisingly, pleasure and habit was not significantly different based on results derived from the SAQ in this study. However, this finding can partly be explained by the questionnaire construction. Patients who are sexually inactive do not complete the questions on pleasure and habit so that inactive patients are not included for the analysis of these scales. Considering sexual inactivity as highly sexually dysfunctioning, we can assume that these results tend to underscore the real level of dysfunction in the BCS group. In view of the SIDI-F pleasure items, herein completed also by inactive women, we found impairments also for pleasure and habit.

The previously reported higher deteriorating effect of chemotherapy and mastectomy [32–35] on SH compared to breast conserving surgery and other adjuvant treatments in or short after the treatment phase seems to be no longer prevalent in the follow-up period. We did not observe an association of any treatment-related variables with SH in BCS. Though corresponding results were observed previously [31], this is in contrast to other established findings. For instance, Raggio and colleagues [25] found mastectomy to have late effects on SH in a smaller sample of BCS up to 7 years post-treatment. Evidence, hence, is somehow inconsistent and needs further elaboration. Larger patient samples would allow the comparison of different chemotherapeutic agents and/or combination treatments regarding their influence on SH which might give further insight into this subject. However, the extent of disease proliferation – indicated by the grade of disease herein – seems to play a role for follow-up SH outcome.

Depressive symptoms, age, and partnership satisfaction seem to be crucial factors for follow-up SH outcome. Low partnership satisfaction and quality are well known to be among the strong factors deteriorating BCS’ SH [31, 36, 37]. This is true also for SH in women without a history of BC [38, 39]. Higher psychological distress and higher age have previously been observed to be associated with reduced SH [26, 40, 41]. Our study findings, thus, confirm established evidence. The role of body image regarding SH outcome seems to be somewhat contradictory. We could not find body image to – though associated – be central to SH problems BCS develop; so did others [42]. However, on the contrary, substantial evidence suggests body image to be among the most important issues for SH after BC [3, 10, 31]. This heterogeneity might be explained by different study groups, study designs, and sample sizes. There are hardly any studies that evaluated the association of body image and sexuality by use of a longitudinal study design with a homogeneously defined baseline and covering a substantial follow-up period. The latter design might contribute to a more precise picture of this association.

Another interesting finding of this study is the reported rate of 60% of HSDD in WNBC. This observation underscores previous estimates of sexual dysfunction as a crucial female problem in general, ranging from 25% to 63% [43]. A BC diagnosis and treatment seems to aggravate this problem of a pretty high pre-diagnosis impairment “level”.

At this point some study limitations have to be discussed. Firstly, we designed the study cross-sectionally. A longitudinal study design including baseline data might have given additional insight into changes of SH over time. Secondly, the use of the SIDI-F as a self-report version has not been validated previously. However, scale reliability and correlation with the widely used and validated PRO measure SAQ was excellent. Subsequently, we can assume this measure to provide validate results. The anonymous completion of questions offered to the patient was considered to contribute to the reduction of potential response bias immanent to PRO assessments. In addition, the potential for selection bias is immanent to studies with a consecutive, unsystematic inclusion procedure. Thirdly, the definition for survivorship used in this study ecompasses the includion of a broad range of patients at different stages post treatment (0.3–17 years), so that the patient comparability might be questioned in this regard. However, we controlled for this factor including time since diagnosis in the analysis showing no impact of this variable on patients’ SH. In addition, this is an issue immanent to survivorship studies in general.

## Conclusion

Our results demonstrate that SH problems persist into BC survivorship and differ significantly from the general population. Sexuality should be regarded as major health care demand in BC survivorship care, as supported also by other authors [44]. A sensibilisation of health care providers towards this subject can be the first step in the improvement of care efforts. Health care providers can break the taboo by addressing SH with the patient as an important survivorship issue in their routine survivorship care/ counselling. The integration of PRO into routine survivorship health care can provide in addition an efficient option to improve the detection of SH problems. Moreover, the routine use of PRO has been observed to facilitate patient-clinician communication and consequently contributes to patient empowerment and patient satisfaction. Awareness and an improved detection of a SH problem can help patients to express their care demands and contribute to targeting treatment efforts. Depending on their SH care demands, patients might be offered some educational information or be referred to a psychologist or gynaecologist specialized on SH for in-depth treatment. Multi-modal treatment options offered by a multi-professional team seem to be most promising for the management of SH problems [44]. However, further reaearch on targeted interventions is required as there are currently hardly any state-of-the-art treatment recommendations for SH for BCS available. Consequently, the treatment of depressive symptoms and partnership problems can contribute to increase not only overall QoL but also BCS’ SH. This is of vital importance with reagard to the sensitive QoL aspect of SH.

## Abbreviations

BC: Breast cancer
BCS: Breast cancer survivors
BIS: Body Image Scale
HADS: Hospital Anxiety and Depresion Scale
HSDD: Hypo-sexual desire disorder
MENQOL: Menopause-Specific Quality of Life Questionnaire
PRO: Patient-reported outcomes
QoL: Quality of life
SAQ: Sexual Activity Questionnaire
SH: Sexual health
SIDI-F: Sexual Interest and Desire Inventory–Female
WNBC: Women from the general population
